# Unusual Dengue Virus 3 Epidemic in Nicaragua, 2009

**DOI:** 10.1371/journal.pntd.0001394

**Published:** 2011-11-08

**Authors:** Gamaliel Gutierrez, Katherine Standish, Federico Narvaez, Maria Angeles Perez, Saira Saborio, Douglas Elizondo, Oscar Ortega, Andrea Nuñez, Guillermina Kuan, Angel Balmaseda, Eva Harris

**Affiliations:** 1 Sustainable Sciences Institute, Managua, Nicaragua; 2 Unidad de Infectología, Hospital Infantil Manuel de Jesús Rivera, Ministry of Health, Managua, Nicaragua; 3 Laboratorio Nacional de Virología, Centro Nacional de Diagnóstico y Referencia, Ministry of Health, Managua, Nicaragua; 4 Centro de Salud Sócrates Flores Vivas, Ministry of Health, Managua, Nicaragua; 5 Division of Infectious Diseases and Vaccinology, School of Public Health, University of California, Berkeley, California, United States of America; University of Rhode Island, United States of America

## Abstract

The four dengue virus serotypes (DENV1–4) cause the most prevalent mosquito-borne viral disease affecting humans worldwide. In 2009, Nicaragua experienced the largest dengue epidemic in over a decade, marked by unusual clinical presentation, as observed in two prospective studies of pediatric dengue in Managua. From August 2009–January 2010, 212 dengue cases were confirmed among 396 study participants at the National Pediatric Reference Hospital. In our parallel community-based cohort study, 170 dengue cases were recorded in 2009–10, compared to 13–65 cases in 2004–9. In both studies, significantly more patients experienced “compensated shock” (poor capillary refill plus cold extremities, tachycardia, tachypnea, and/or weak pulse) in 2009–10 than in previous years (42.5% [90/212] vs. 24.7% [82/332] in the hospital study (p<0.001) and 17% [29/170] vs. 2.2% [4/181] in the cohort study (p<0.001). Signs of poor peripheral perfusion presented significantly earlier (1–2 days) in 2009–10 than in previous years according to Kaplan-Meier survival analysis. In the hospital study, 19.8% of subjects were transferred to intensive care, compared to 7.1% in previous years – similar to the cohort study. DENV-3 predominated in 2008–9, 2009–10, and 2010–11, and full-length sequencing revealed no major genetic changes from 2008–9 to 2010–11. In 2008–9 and 2010–11, typical dengue was observed; only in 2009–10 was unusual presentation noted. Multivariate analysis revealed only “2009–10” as a significant risk factor for Dengue Fever with Compensated Shock. Interestingly, circulation of pandemic influenza A-H1N1 2009 in Managua was shifted such that it overlapped with the dengue epidemic. We hypothesize that prior influenza A H1N1 2009 infection may have modulated subsequent DENV infection, and initial results of an ongoing study suggest increased risk of shock among children with anti-H1N1-2009 antibodies. This study demonstrates that parameters other than serotype, viral genomic sequence, immune status, and sequence of serotypes can play a role in modulating dengue disease outcome.

## Author Summary

Dengue is the most prevalent mosquito-borne viral disease affecting humans worldwide. The four dengue virus serotypes (DENV1–4) cause Dengue Fever and more severe life-threatening syndromes. In 2009, Nicaragua experienced the largest dengue epidemic in over a decade. In a hospital-based study and community-based prospective cohort study of pediatric dengue in the capital, Managua, we observed unusual clinical presentation of dengue. Significantly more patients experienced “compensated shock” (poor capillary refill plus cold extremities, rapid heartbeat, elevated respiratory rate, and/or weak pulse) in 2009–10 than in previous years. These signs of poor peripheral perfusion presented significantly earlier and more children were transferred to intensive care in 2009–10 than in previous years. DENV-3 was the predominant serotype in Managua in 2008–9, 2009–10 and 2010–11, but full-length sequencing revealed no major genetic changes. In 2008–9 and 2010–11, typical dengue was observed; only in 2009–10 was unusual presentation noted. Since pandemic influenza A H1N1-2009 overlapped with the dengue epidemic in Nicaragua, we hypothesize that prior influenza A H1N1-2009 infection may have modulated subsequent DENV infection, and preliminary results appear to support this hypothesis. This study demonstrates that parameters other than DENV serotype, viral genomic sequence, immune status, and sequence of serotypes can play a role in modulating dengue disease.

## Introduction

Dengue is an increasing public health problem in tropical and sub-tropical regions, with tens of millions of cases estimated to occur annually [1]. The four serotypes of dengue virus (DENV-1–4), a mosquito-borne *Flavivirus*, cause a range of clinical manifestations, from undifferentiated illness and classic Dengue Fever, to more severe syndromes characterized by plasma leakage, shock, and death, referred to as Dengue Hemorrhagic Fever and Dengue Shock Syndrome (DHF/DSS) [2]. While serotype and dengue immune status have been shown to affect severity of disease [3], [4], [5], [6], many of the epidemiologic and clinical variations in the presentation of dengue remain poorly understood.

Dengue transmission has increased dramatically in Nicaragua and much of the rest of the Americas in the past three decades. All four serotypes now circulate in Nicaragua, though unlike Asia, where dengue is hyperendemic [3], [7], one serotype usually predominates each year [8], [9], [10], [11], [12]. The dengue season starts some months after the first rains, and typically lasts from August to January. In 2004–2008, several thousand cases of laboratory-confirmed dengue were reported annually by the National Epidemiologic Surveillance program, though actual numbers of cases are suspected to be much higher [13]. Approximately 2–10% of laboratory-confirmed dengue cases were the more severe dengue hemorrhagic fever (DHF) or dengue shock syndrome (DSS), and less than 1% resulted in death (A. Nuñez and A. Balmaseda, unpublished data).

In 2009–10, Nicaragua experienced one of the largest dengue epidemics since the virus was reintroduced into the country in the 1980s, with approximately 3 times as many laboratory-confirmed cases reported by national authorities and documented in our studies than in the previous five years. The same serotype, DENV-3, was responsible for the majority of cases in the 2008–9, 2009–10, and 2010–11 seasons, but caused an atypical clinical presentation only in 2009–10, characterized primarily by early signs of poor peripheral perfusion and what is designated “compensated shock”. Here we present epidemiologic and clinical data from two on-going prospective studies of pediatric dengue in Managua, Nicaragua, that define the characteristics of the epidemic and begin to investigate possible explanations.

## Materials and Methods

### Cohort Study

The Pediatric Dengue Cohort Study is a community-based prospective study that was initiated in 2004 in the low- to middle-income District II of Managua, Nicaragua, close to Lake Nicaragua, in which most residents attend the local municipal health center, the Health Center Socrates Flores Vivas (HCSFV). Children aged two to nine years old living in the catchment area of the HCSFV were initially enrolled in August–September 2004, and new 2-year olds have been enrolled each year since then. Children are withdrawn from the study when they reach 15 years of age. Study design and methods have been previously described [14].

Participants are encouraged to present at the first sign of illness to the HCSFV, where study physicians screen them for signs and symptoms of dengue and presence of warning signs for severity using a standardized data collection form. Subjects are followed daily during the acute phase of illness by physicians at the HCSFV or via home visits by study nurses. Acute and convalescent (14 days after onset of fever) blood samples are drawn for virological, serological, and molecular biological testing for dengue, and additional blood samples are drawn every other day during the acute phase of illness for Complete Blood Count (CBC) and blood chemistry tests as warranted. Participants are transferred to the National Pediatric Reference Hospital in Managua (Hospital Infantil Manuel de Jesús Rivera, HIMJR) if warning signs or risk factors are present (see below). Trained study physicians and nurses also collect data using standardized forms at the HIMJR. Additionally, each year in July–August, a healthy blood sample is drawn from all subjects. Sera from consecutive annual samples are tested for the presence of anti-DENV antibodies to identify silent transmission during the year and to determine dengue immune status.

### Hospital-Based Study

Beginning in 1998, a prospective study of pediatric dengue has been carried out in the Infectious Disease Ward of the HIMJR to study clinical, immunological and viral risk factors for severe dengue [9], [10], [15], [16], [17], [18]. This report focuses on data from August 2005 through January 2010. Children between six months and 14 years of age with suspected dengue (<7 days of illness) and who are not actively enrolled in the concurrent cohort study are eligible to participate in the hospital study. Both in-patient and out-patient subjects are enrolled each year during the dengue season (August–January) and followed clinically through the acute phase of illness.

Upon enrollment, a medical history is taken and a complete physical exam is performed. Clinical data, including vital signs, symptoms, fluid balance and treatment, are recorded daily on standardized data collection forms during hospitalization or through daily ambulatory follow-up visits by the same team of study physicians and nurses responsible for care of hospitalized cohort study participants. Acute blood samples are taken daily for CBC and serological, virological, and molecular biological testing for DENV infection, and ultrasound and/or X-rays are performed daily. Participants requiring more intensive therapies are transferred to the intensive care unit (ICU). A convalescent-phase blood sample (two weeks after presentation to hospital) is also collected.

### Ethics Statement

The protocols for both studies were reviewed and approved by the Institutional Review Boards (IRB) of the University of California, Berkeley, and of the Nicaraguan Ministry of Health; additionally, the cohort study protocol was reviewed and approved by the IRB of the International Vaccine Institute in Seoul, South Korea. Parents or legal guardians of all subjects in both studies provided written informed consent, and subjects 6 years of age and older provided assent.

### Laboratory Assays

Acute-phase serum samples are tested for DENV RNA using a nested reverse transcriptase–polymerase chain reaction (RT-PCR) directed to the capsid gene, which also permits identification of serotype [19]. Samples positive by RT-PCR are processed for viral isolation by inoculation onto *Ae. albopictus* C6/36 cells [20]. Paired acute- and convalescent-phase samples are tested for anti-DENV IgM antibodies using an in-house IgM capture ELISA [21] and for total anti-DENV antibodies by Inhibition ELISA [11], [22]. The presence of anti-influenza A H1N1 2009 antibodies in paired acute- and convalescent-phase samples from 2009–10 DENV-positive hospital subjects was determined using the hemagglutination inhibition assay [23]. The antigen used was prepared using Nicaraguan strain A/Managua/2339.03 09-H1N1-SW and is specific for the H1N9 2009 strain of Influenza A (S. Saborio and A. Balmaseda, unpublished data).

### Sequencing and Phylogenetic Analysis

DENV isolates were sequenced at the Broad institute using a combination of Sanger sequencing and high-throughput (454/Roche) pyrosequencing. Sequences were aligned using Muscle [24], with parameters optimized for maximum accuracy (i.e., no limit was specified for runtime or iteration count). Aligned sequences were clustered using phyML [25] with the following parameters: (a) substitution model = HKY85, (b) number of substitution categories = four, (c) proportion of invariant sites = zero, and (d) estimated values for equilibrium frequency, transition/transversion ratio and gamma shape parameter.

### Definitions

In these studies, a suspected dengue case was considered positive if it met one of the following criteria: DENV was isolated; DENV RNA was detected through RT-PCR; seroconversion of DENV-specific IgM was detected in paired acute- and convalescent-phase samples; or antibody titer by Inhibition ELISA demonstrated a 4-fold or greater increase in titer between acute and convalescent sera [9]. Primary DENV infections were those in which acute antibody titer was <10 or convalescent antibody titer was <2,560 and secondary infections were those in which antibody titer was ≥10 (acute) or ≥2,560 (convalescent) as determined by Inhibition ELISA [12]. Hospital cases from 2009–10 were considered positive for anti-influenza A H1N1 2009 antibodies if the HI titer was ≥20 in acute samples or if seroconversion or a ≥4-fold increase in HI titer was observed in paired acute and convalescent samples.

In 2004–9, cohort subjects presenting to the HCSFV were referred to the HIMJR, and hospital study participants were hospitalized, if they presented any of the following warning signs: persistent vomiting; moderate-to-severe dehydration; signs or symptoms of shock; abdominal pain; breathing difficulties; moderate-to-severe hemorrhagic manifestations; neurological manifestations; thrombocytopenia (platelet count ≤100,000 platelets/µL); or hematocrit ≥20% of normal value for age and sex. Children with known risk factors were also referred to the hospital, including those who were overweight and/or under one year of age. During the 2009–10 dengue epidemic, national health authorities mandated primary care physicians to refer all suspected dengue cases with the above signs of alarm to the hospital, regardless of clinical laboratory results, and hospitals to admit all referrals from health centers for observation.

All laboratory-confirmed cases without signs of severity were classified as dengue fever (DF), including cases presenting as undifferentiated fever. Severity was categorized according to the 1997 WHO guidelines [2]: Dengue Hemorrhagic Fever (DHF; hemorrhagic manifestations, platelet count ≤100,000 platelets/µL, and evidence of plasma leakage) and Dengue Shock Syndrome (DSS; DHF with circulatory disturbance evidenced by hypotension for age or narrow pulse pressure accompanied by clinical signs of shock). In addition, included here among categories of severe dengue are “Dengue with Signs Associated with Shock” (DSAS; DF with hypotension or narrow pulse pressure plus one or more of the following: capillary refill >2 seconds, cold extremities, or weak pulse) [9] and “Dengue Fever with Compensated Shock (DFCS; DF with capillary refill >2 sec plus cold extremities, poor or impalpable pulse, tachycardia, and/or tachypnea (increased breathing rate for age) on the same day). While clinical classifications by study physicians were assigned at discharge, here we present classifications determined by applying algorithms for DHF, DSS, DSAS and DFCS to clinical data *ex post facto*. Separate from DFCS as a disease classification, patients were considered to have experienced “compensated shock” if they presented with capillary refill >2 sec plus cold extremities, poor or impalpable pulse, tachycardia, and/or tachypnea on the same day; this state could or could not evolve into hypotensive shock depending on treatment and patient response. Thus, cases classified as DHF, DSS or DSAS may have experienced compensated shock during the course of illness.

Delayed capillary refill, a sign of vasoconstriction and low blood volume, was the key clinical criteria for compensated shock, along with signs of circulatory distress, including cold extremities. In this study, study physicians and nurses used standard techniques of observation. Capillary refill time was determined by the clinician by briefly pressing on the pad of the patient's index finger and counting the number of seconds before normal color returned. Cold extremities is defined as lower than normal skin temperature and was observed by the clinician by touching with the dorsal side of the hand the patient's extremities, most commonly the plantar side of the foot. Cold and clammy skin, which is included in the variable cold extremities here, was also observed by palpating the plantar region of the inferior extremities with the dorsal side of the hand to sense coldness, clamminess, and sweat.

### Data Analysis

Demographic and clinical characteristics of dengue cases in both studies are presented by dengue season, defined in the year-round cohort study as July 1–June 30 of each year in years 2005–2010 and August 1, 2004–June 30, 2005 in the first year of the study. In the hospital-based study, the dengue season is defined as August 1–January 31, months corresponding to the highest incidence of symptomatic DENV infection, when study enrollment occurs (clinical follow-up continues through the month of February each year). Total and age-stratified incidence in the cohort study were calculated using laboratory-confirmed dengue cases and the number of participants active at the time of the annual healthy blood sample collected in July of each year.

Univariate and bivariate analyses were performed comparing cases during the 2009–10 dengue season to cases in previous dengue seasons. For bivariate comparison of cases across seasons, chi squared and Fisher's exact tests were applied to categorical variables, while the t-test and non-parametric Mann-Whitney test were applied to continuous variables. Kaplan-Meyer survival analysis of symptom presentation by day was performed controlling for early presentation, defined as presenting to hospital or health center in the first 3 days after onset of symptoms. Log-rank tests were performed to compare survival curves, and Cox regression models were created to determine risk factors associated with “compensated shock”, poor capillary refill, and cold extremities, controlling for dengue season, serotype, immune response, age, sex, and early presentation. Dummy variables were created for categorical variables with more than 2 values, utilizing as the reference the variable that presented least risk. Risk factors associated with DFCS as a disease classification were estimated using generalized linear model (GLM) multivariate analysis, controlling for the same variables as above. Confidence levels of 95% were used. In both studies, data were stored in Microsoft Access 2003, and statistical analyses were performed using Intercooled Stata, Version 9 (StataCorp).

## Results

### General Characteristics of the 2009–10 Epidemic

**Figure 1:**
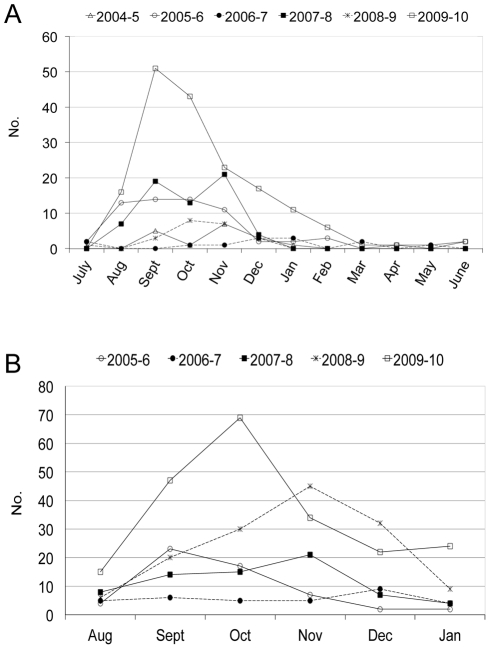
Dengue epidemics in prospective pediatric studies in Managua, Nicaragua, 2004–10. *A*, Number of confirmed dengue cases by month, cohort study, 2004–10; *B*, number of confirmed dengue cases by month, hospital study, 2005–10.

Between August 2009 and June 2010, 170 laboratory-confirmed, symptomatic cases of dengue were identified among the 3,711 active cohort participants, with 97.7% occurring between August and February (Fig. 1A). Overall, a dengue case incidence of 4.6% was observed, compared to 0.4–1.8% in 2004–9 (between 13 and 65 cases each dengue season, Table 1). In the concurrent hospital-based study, 212 (54% of enrolled participants) dengue cases were confirmed among 396 study participants during the enrollment period spanning August 2009–January 2010 (Fig. 1B). While the design of the hospital study does not allow calculation of incidence, surveillance records indicate that 5–10 times more suspected dengue cases presented to the HIMJR in 2009–10 than in previous study years (A. Balmaseda and M.A. Perez, unpublished data). The greatest numbers of cases were observed in September and October in both studies (Fig. 1A and B). As in previous years, dengue was distributed evenly by gender (50.0% were female in both studies), and cases were encountered in subjects of all ages. The mean age of dengue cases in the cohort was greater in 2009–10 due to aging of the cohort, which by then extended to 13–14 years of age; no such age variation is seen in the hospital study, where subject ages were constant through all 5 years of the study.

**Table 1:** Characteristics of confirmed dengue cases, Cohort Study, 2004–10, and Hospital Study, 2005–2010.

	Cohort Study			Hospital Study		
	2004–9	2009–10	p-value^5^	2005–9	2009–10	p-value^5^
	n (%)	n (%)		n (%)	n (%)	
**Symptomatic dengue cases (% annual incidence)** 1	181 (0.4–1.8)	170 (4.6)		332	212	
**Sex**			0.641			1.000
**Female**	86 (47.5)	85 (50.0)		166 (50.0)	106 (50.0)	
**Male**	95 (52.5)	85 (50.0)		166 (50.0)	106 (50.0)	
**Age, years (mean, SE)**	7.2 (2.6)	8.2 (3.0)	0.002	8.4 (4.8–11.5)	8.6 (5.2–10.8)	0.8831
**Serotype** 2						
**DENV-1**	35 (21.5)	18 (10.7)	0.007	35 (11.8)	10 (5.1)	0.011
**DENV-2**	106 (65.0)	9 (5.4)	<0.001	150 (50.5)	11 (5.6)	<0.001
**DENV-3**	19 (11.7)	141 (83.9)	<0.001	112 (37.7)	175 (88.8)	<0.001
**DENV-4**	1 (0.6)	0 (0.0)	----	0 (0.0)	0 (0.0)	----
**DENV 1 & 4**	1 (0.6)	0 (0.0)	----	0 (0.0)	0 (0.0)	----
**DENV 1 & 2**	1 (0.6)	0 (0.0)	----	0 (0.0)	0 (0.0)	----
**DENV-3 & 4**	0 (0.0)	0 (0.0)	----	0 (0.0)	1 (0.5)	----
**Immune Response** 3			0.424			<0.001
**Primary**	77 (43.3)	78 (47.6)		109 (34.1)	107 (52.2)	
**Secondary**	101 (56.7)	86 (52.4)		211 (65.9)	98 (47.8)	
**Day of presentation after onset of symptoms (mean, SD)**	2.2 (1.0)	2.1 (0.9)	0.9	4.6 (1.1)	3.5 (1.2)	<0.001
**Care**						
**Out-patient**	157 (86.7)	91 (53.5)	<0.001	58 (17.5)	29 (13.7)	0.239
**In-patient**	22 (12.2)	60 (35.3)	<0.001	258 (77.7)	141 (66.5)	0.004
**Intensive care** 4	2 (1.1)	19 (11.2)	<0.001	16 (7.1)	42 (19.8)	<0.001
**Classification**						
**Dengue Fever (DF)**	161 (88.9)	126 (74.1)	<0.001	164 (49.4)	104 (49.1)	0.938
**Dengue Fever with Compensated Shock (DFCS)**	4 (2.2)	15 (8.8)	0.005	23 (6.9)	50 (23.6)	<0.001
**Dengue w/Signs Associated w/Shock (DSAS)**	3 (1.7)	10 (5.9)	0.032	16 (4.8)	28 (13.2)	<0.001
**Dengue Hemorrhagic Fever (DHF)**	9 (5.0)	12 (7.1)	0.409	82 (24.7)	24 (11.3)	<0.001
**Dengue Shock Syndrome (DSS)**	4 (2.2)	7 (4.1)	0.303	47 (14.2)	6 (2.8)	<0.001

1 Incidence has only been calculated in the cohort study, in which between 3,497 and 3753 subjects were active in each of the six study years, corresponding to symptomatic dengue incidence of 0.48% in 2004–5, 1.82% in 2005–6, 0.37% in 2006–7, 1.82% in 2007–8, 0.59% in 2008–9 and 4.61% in 2009–10.

2 In the cohort study, serotype is known for 14 cases in 2004–5, 56 cases in 2005–6, 10 cases in 2006–7, 62 cases in 2007–8, 21 cases in 2008–9 and 170 cases in 2009–10. In the hospital study, serotype was identified in 46, 43, 69, 139 and 197 cases in years 2005–6 through 2009–10, respectively.

3 In the cohort study, immune response is indeterminate for one case in each year, 2006–7, 2007–8 and 2008–9, and in 6 cases in 2009–10. In the hospital study, immune response is known in 55, 46, 72, 147 and 203 cases in years 2005–6 through 2009–10, respectively.

4 In 2005–6 and 2006–7, cases requiring intensive care were not documented in the hospital study.

5 p-values were calculated using the Chi-square tests except for mean age and mean day of presentation, for which Mann-Whitney t-tests were applied.

DENV-3 caused 88.8% (hospital study) and 83.9% (cohort) of cases in 2009–2010. DENV-1 and -2 were also responsible for cases in both studies in 2009–10, and one subject in the hospital study was positive for both DENV-3 and DENV-4 (Table 1). Roughly half of dengue cases in both studies experienced a primary immune response (47.6% in the cohort study and 52.2% in the hospital study). More secondary cases were observed in both studies prior to 2008 due to the dominant circulation of DENV-2 in 2005–8, although only in the hospital study was this difference significant (p<0.001). A larger proportion of DENV infections in 2009–10 presented symptomatically (1 symptomatic case for every 1.2 inapparent DENV infections, as measured via the annual healthy blood sample), as compared to 2008–9, when DENV-3 also predominated (1∶9.5 symptomatic to inapparent DENV infection ratio; p<0.001).

### Clinical Presentation

**Figure 2:**
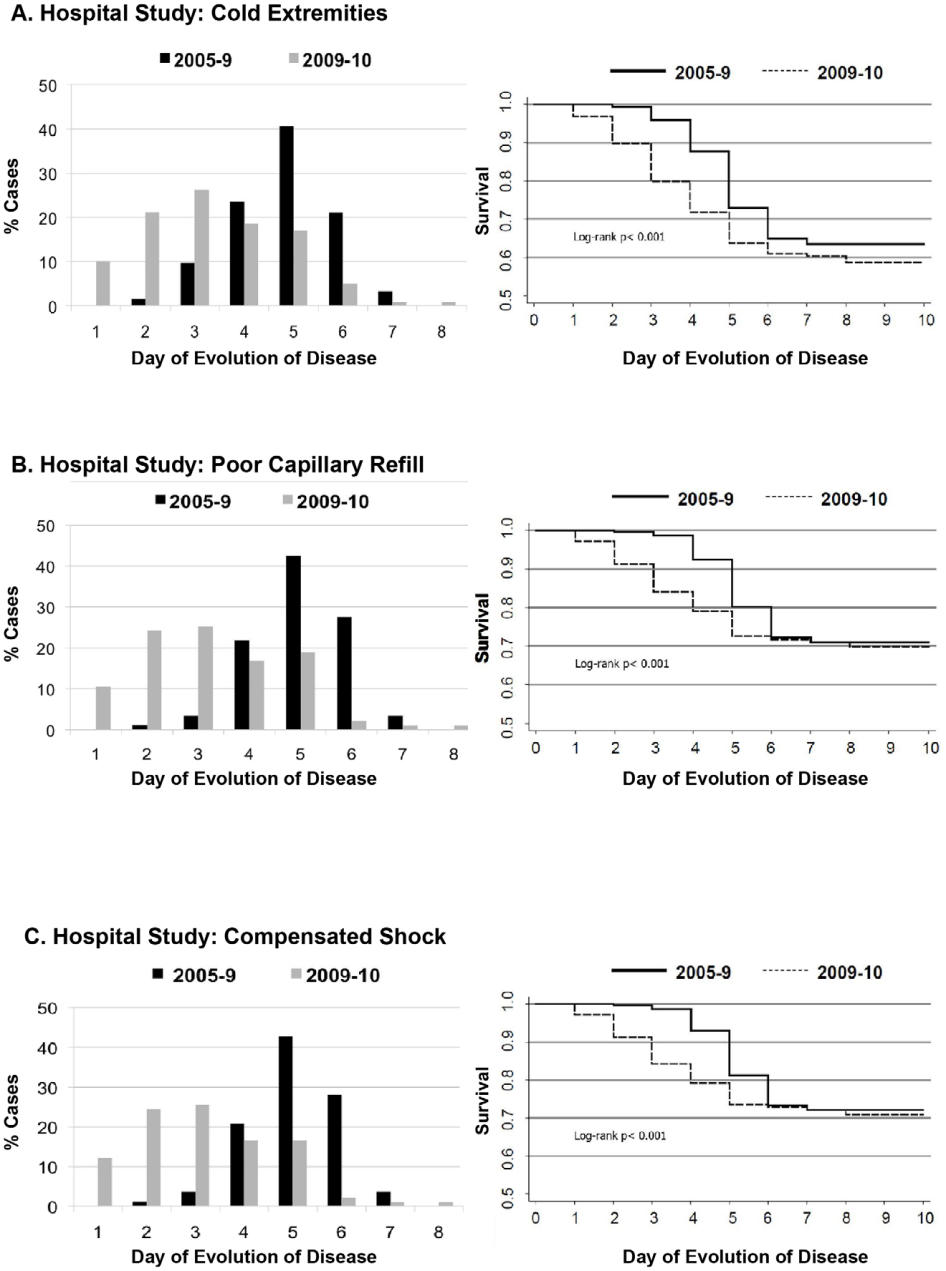
Presentation of signs of poor peripheral perfusion in hospital study dengue cases, 2005–9 vs. 2009–10. *A*, cold extremities, *B*, poor capillary refill (>2 sec), and *C*, compensated shock. Left panel, frequency of presentation by day; right panel, Kaplan-Meier survival function adjusted for early presentation (days 1–3 after onset of fever).

**Figure 3:**
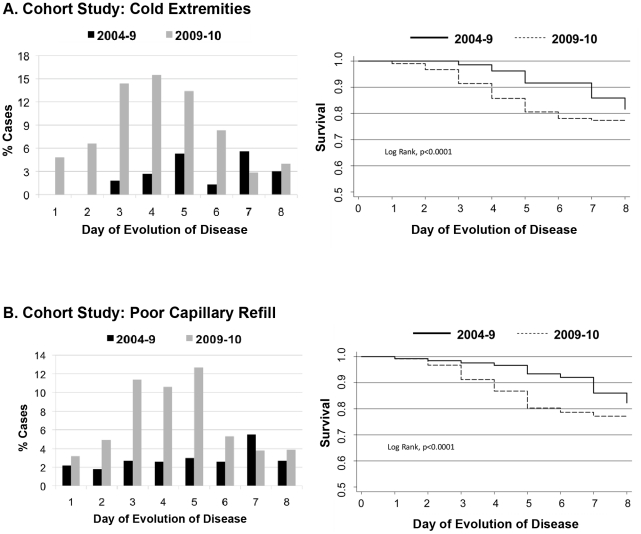
Presentation of signs of poor peripheral perfusion in the dengue cohort study, 2004–9 vs. 2009–10. *A*, cold extremities and *B*, poor capillary refill (>2 sec). Left panel, frequency of presentation by day; right panel, Kaplan-Meier survival function adjusted for early presentation (days 1–3 after onset of fever).

In both studies, the clinical presentation of dengue was markedly different in 2009–10 than in previous years (Table 2). Unique to the 2009–10 season was the significantly more frequent presentation of poor peripheral perfusion, specifically poor capillary refill (>2 seconds) and cold extremities. Significantly more patients experienced “compensated shock” (poor capillary refill plus cold extremities, tachycardia, tachypnea, and/or weak pulse) in 2009–10 than in 2005–8 (42.5% [90/212] vs. 24.7% [82/332] in the hospital study (p<0.001) and 17.0% [29/170] vs. 2.2% [4/181] in the cohort study (p<0.001)) (Table 2). In Kaplan-Meier survival analysis, poor capillary refill and cold extremities presented significantly earlier (1–2 days) in both studies when controlling for day of presentation (log rank p<0.001 in both studies, Figs. 2A and B, 3A and B). Kaplan-Meier survival analysis of “compensated shock” as an entity comprising these signs naturally yielded similar results (Fig. 2C). When controlling for serotype, immune response and early presentation in a Cox regression model, hospital study dengue cases in 2009–10 had 2.80 times more risk of presenting poor capillary refill (95% Confidence Intervals (CI) 1.85–4.23; p<0.001), 2.13 times more risk of presenting cold extremities (95%CI 1.52–3.00; p<0.001), and 2.81 times more risk of presenting “compensated shock” (95%CI 1.85–4.27; p<0.001); performing the same analysis among cohort cases produced similar results (Table 3).

**Table 2:** Clinical signs and symptoms of confirmed dengue cases in 2009–10 compared to previous years, 2004–10.

	Cohort Study			Hospital Study		
	2004–9	2009–10	p-value	2005–9	2009–10	p-value
	n = 181 (%)	n = 170 (%)		n = 332 (%)	n = 212 (%)	
**Hemorrhagic manifestation** 1	96 (53.0)	119 (70.0)	0.001	260 (78.3)	180 (84.1)	0.057
**Mucosal bleeding**	28 (15.5)	49 (28.8)	0.003	61 (18.3)	37 (17.4)	0.785
**Positive tourniquet test**	86 (47.5)	97 (57.1)	0.07	156 (47.0)	125 (59.0)	0.006
**Petechiae**	51 (28.2)	79 (46.5)	<0.001	250 (75.3)	167 (78.8)	0.350
**Rash**	65 (35.9)	85 (50.0)	0.008	302 (91.0)	196 (92.4)	0.543
**Platelets ≤100,000/mm^3^**	21 (11.7)	31 (19.0)	0.06	174 (52.4)	56 (26.5)	<0.001
**Leukopenia (WBC ≤5,000/mm^3^)**	117 (65.0)	142 (87.1)	<0.001	253 (76.2)	192 (90.6)	<0.001
**Cold extremities**	14 (7.7)	59 (34.7)	<0.001	123 (37.1)	118 (55.6)	<0.001
**Capillary refill >2 seconds**	12 (6.6)	50 (29.4)	<0.001	87 (26.2)	94 (44.34)	<0.001
**Compensated shock**	4 (2.2)	29 (17)	<0.001	82 (24.7)	90 (42.5)	<0.001

1 Hemorrhagic manifestations do not include laboratory values and include any of the following clinical signs and symptoms: petechiae, rash, positive tourniquet test, bruising, hematoma, hemoptysis, epistaxis, gingivorrhagia, melena, hematemesis, hematuria, subconjunctival hemorrhage, vaginal hemorrhage, hypermenorrhea and excessive bleeding at puncture site.

**Table 3:** Year 2009–10 as most significant risk factor in Cox regression models of compensated shock, 2004–10.

	Cohort Study			Hospital Study		
	Hazard Ratio for Year 2009–10^1^	95% CI	p-value	Hazard Ratio for Year 2009–10^1^	95% CI	p-value
**Cold extremities**	3.16	1.40–7.15	0.006	2.13	1.52–3.00	<0.001
**Capillary refill >2 seconds**	2.93	1.21–7.06	0.017	2.80	1.85–4.23	<0.001
**Compensated shock**	—	—		2.81	1.85–4.27	<0.001

1 Cox regression models were created to determine risk factors associated with “compensated shock”, poor capillary refill, and cold extremities, controlling for dengue season, serotype, immune response, age, sex, and early presentation. Year 2009–10 emerged as the most significant risk factor in all models, with values as indicated.

In addition, thrombocytopenia was seen less frequently in 2009–10 than in previous years in the hospital study (26.5% vs. 52.4% in 2005–9, p<0.001, Table 2), although there was a trend toward more thrombocytopenia in the cohort study in 2009–10. Clinical manifestations of hemorrhage were more frequently observed in both studies in 2009–10, though this difference was only significant in the cohort study (70.0% vs. 53.0%, p<0.01, and 84.1% vs. 78.3%, p>0.05 in the cohort and hospital studies, respectively).

### Severity and Classification

In both studies, more dengue-positive children required intensive care in 2009–10 than in previous years. In the cohort study, 11.2% (19/170) of all cases were admitted to the ICU, while only 1.1% (2/181) cases were transferred to intensive care in 2004–9 (p<0.001). In the hospital study, 19.8% (42/212) of patients with confirmed dengue were transferred to intensive care in 2009–10 compared to 7.1% (16/225) in 2007–9 (p<0.001; ICU data is available in the hospital study beginning in 2007). This increase in ICU transfer was due to markedly greater presentation of “compensated shock” in 2009–10 than in previous years. Children with signs of poor peripheral perfusion (“compensated shock”) were administered crystalloid fluid IV and if unresponsive, then colloids were given. When signs of poor peripheral perfusion persisted, children were transferred to the ICU. In the hospital study, of subjects presenting “compensated shock”, 96% (86/90) in 2009–10 received IV fluid therapy, and 40.7% (35/86) of those given liquids were transferred to the ICU. In 2005–9, 83% (68/82) of patients with “compensated shock” received IV fluids, of which 23% (15/68) were transferred to the ICU (during 2007–9). The early administration of IV crystalloid and colloid fluids may well have contributed to preventing progression to hypotensive shock. For instance, in the hospital study, only 30% (27/90) of children experiencing “compensated shock” progressed to hypotensive shock; this could explain the reduced numbers of DHF/DSS noted in 2009–10. In 2005–9, 51% (42/82) of children with “compensated shock” progressed to hypotensive shock.

**Figure 4:**
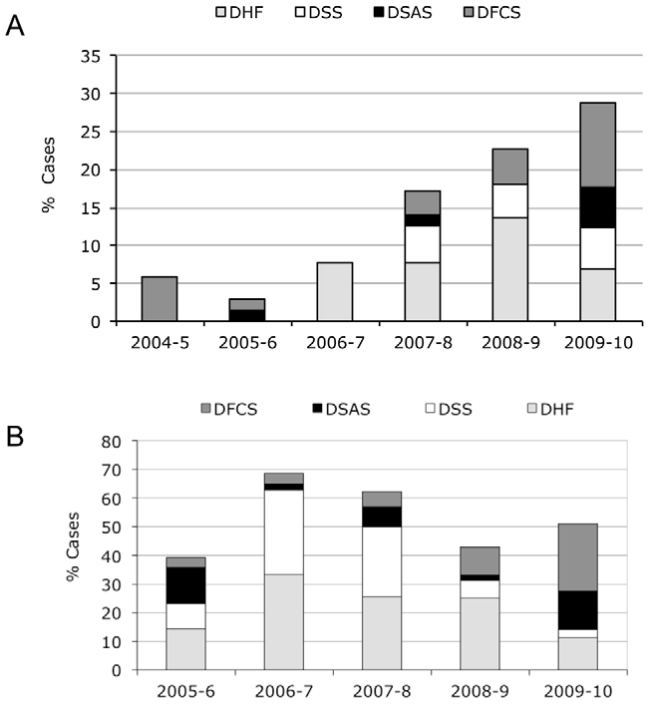
Classification of severity among confirmed dengue cases by year. Dengue cases were classified according to WHO classification (Dengue Hemorrhagic Fever and Dengue Shock Syndrome), Dengue Fever with Compensated Shock (DFCS), and Dengue with Signs Associated with Shock (DSAS), in *A*, cohort study, 2004–10, and *B*, hospital study, 2005–10.

When children who experienced “compensated shock” did not progress to hypotensive shock, they were classified as “Dengue Fever with Compensated Shock” (DFCS). Significantly more cases of DFCS were observed in 2009–10 as compared to 2004–9 in the cohort study (15/170 [8.8%] vs. 4/181 [2.2%]) and in the hospital study (50/212 [23.6%] vs. 23/332 [6.9%]) (Table 1 and Fig. 4). Conversely, in the hospital study, significantly less DHF and DSS were seen in 2009–10 (11.3% and 2.8%, respectively) compared to 2005–9 (24.7% and 14.2%, respectively, p<0.001, Table 1 and Fig. 4B). However, more DSAS cases were seen in 2009–10 (28/212; 13.2%) than in 2005–9 (16/332; 4.8%). Interestingly, in 2009–10, DFCS, DSAS, and DSS cases were comprised of equivalent numbers of primary and secondary DENV infections.

When multivariate analysis was performed to examine risk factors associated with classification as DFCS as compared to all other dengue cases, controlling for immune status, year 2009–10 versus 2005–9, serotype, sex, and age (<5 years old), only year of study 2009–10 emerged as significant (RR 3.42, 95%CI 1.67–7.01; p<0.001; Table 4). Likewise, in multivariate analysis controlling for the same variables as above, only study year 2009–10 was a significant risk factor for DFCS as compared to uncomplicated dengue fever (RR 3.02, 95%CI 1.64–5.59; p<0.001) or for shock (both compensated and hypotensive) as compared to non-shock dengue cases (RR 2.42, 95%CI 1.62–3.11; p<0.001).

**Table 4:** Multivariate analysis of risk factors for DFCS compared to other dengue cases, Hospital Study, 2005–10.

	Relative Risk	95% CI	p-value
**Year 2009–10** 1	3.42	1.67–7.01	0.001
**Secondary immune response**	1.00	0.58–1.72	0.997
**DENV-1**	2.06	0.79–5.40	0.141
**DENV-2**	1.00	Reference value	—
**DENV-3**	1.24	0.53–2.90	0.613
**Age <5 years**	1.12	0.60–2.10	0.716
**Female**	0.78	0.49–1.25	0.314
**Early presentation**	1.02	0.61–1.73	0.912

1 The reference group is years 2005–9.

### DENV-3 Subanalysis in 2005–9, 2009–10 and 2010–11

**Figure 5:**
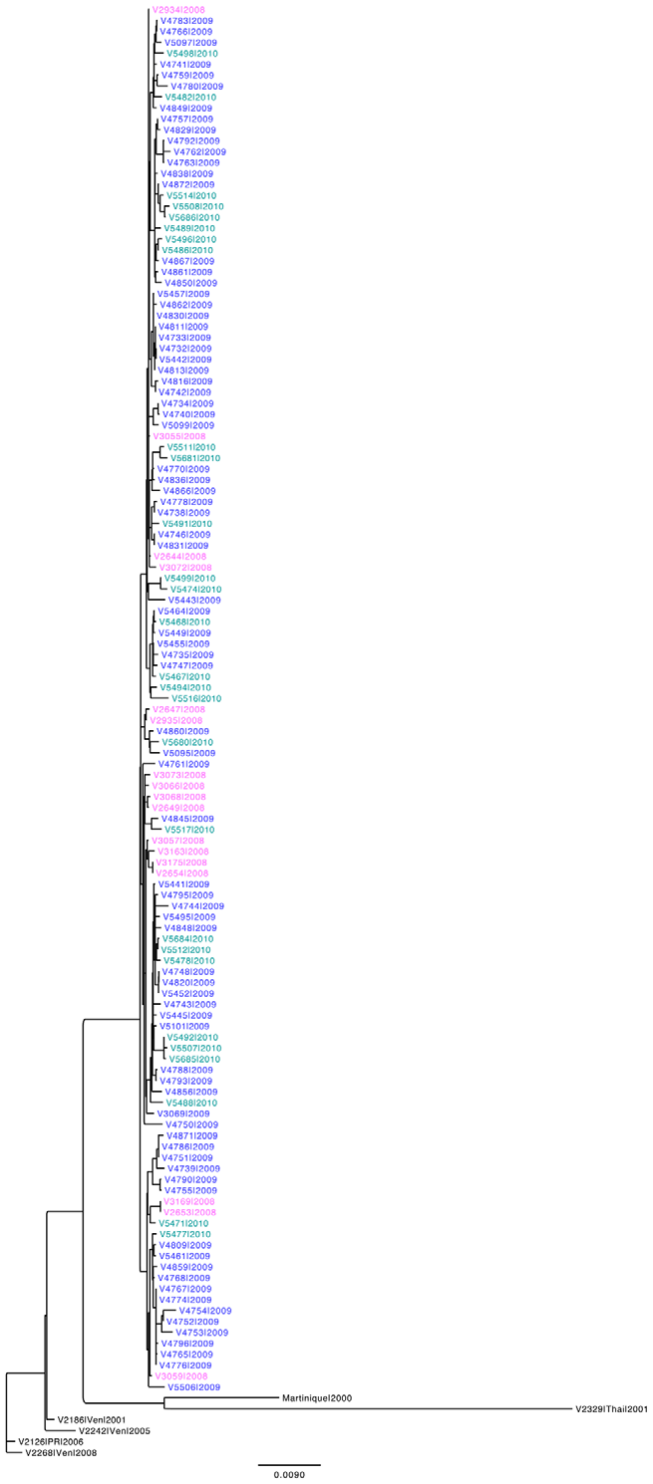
Phylogenetic analysis of Nicaraguan DENV-3 sequences from 2008–2010. Seventeen, 82, and 28 sequences from 2008 (pink), 2009 (blue) and 2010 (teal), respectively, were aligned using Muscle and clustered using phyML. Six isolates from Venezuela (“Ven”), Martinique (“Martinique”), Thailand (“Thai”) and Puerto Rico (“PR”) from the Asian-American genotype are also shown to root the tree and demonstrate similarities among the Nicaraguan isolates.

**Figure 6:**
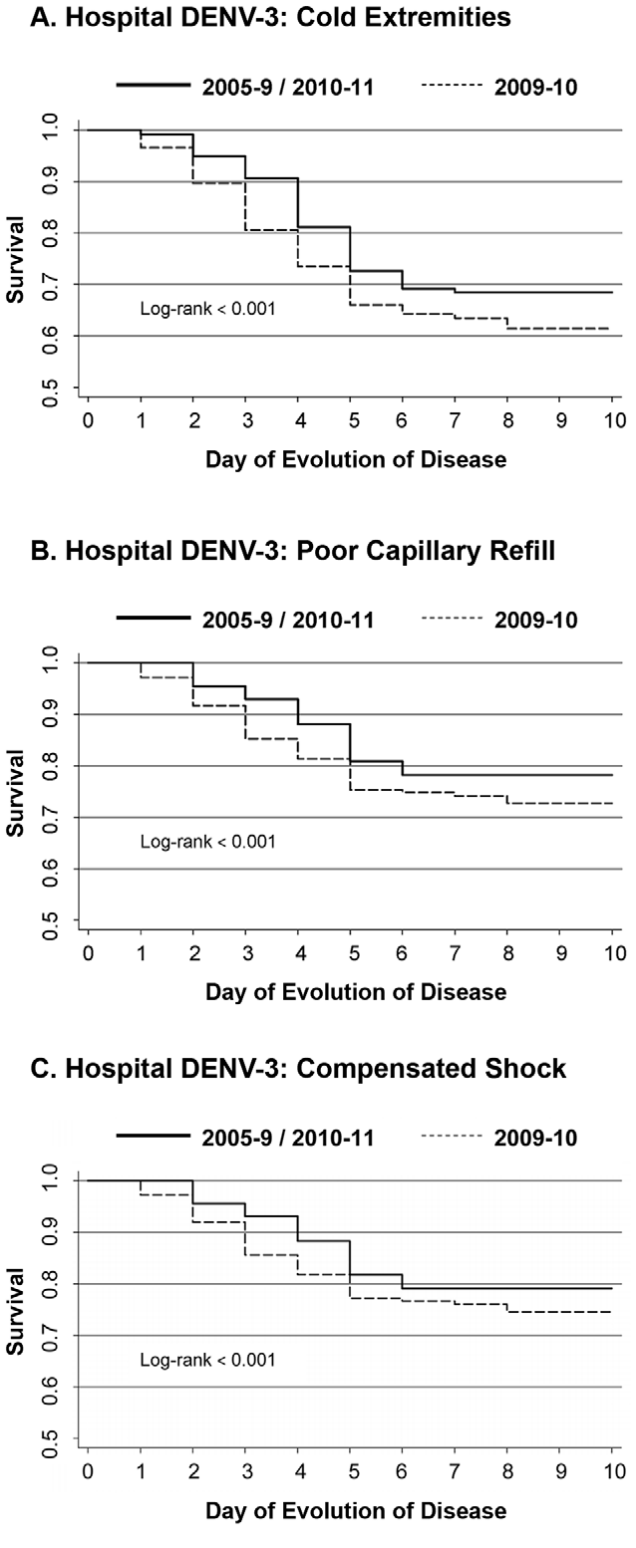
Presentation of signs of poor peripheral perfusion in DENV-3 cases from the hospital study, 2009–10 vs. 2005–9/2010–11. Kaplan-Meier survivor function was adjusted for early presentation (days 1–3 after onset of fever) for *A*, cold extremities, *B*, capillary refill >2 seconds, and *C*, compensated shock.

Beginning in 2008–9 and continuing through 2010–11, DENV-3 caused the majority of dengue cases in both the cohort and hospital studies, differentiating these years from the previous study years in which DENV-2 predominated. In 2008–9, 2009–10 and 2010–11, DENV-3 was responsible for between 74 and 97% of cases in the hospital study, and between 80 and 90% of cases in the cohort study. We sequenced the full-length genome of 17 DENV-3 strains from the 2008–9 dengue season, 82 strains from the 2009–10 dengue season, and 28 samples from the 2010–11 dengue season; phylogenetic analysis revealed no changes in genotype or clade (Fig. 5). We then performed a sub-analysis among DENV-3 cases in the hospital study to control for the effect of serotype on clinical presentation. Compared to cases in 2005–9 and 2010–11, more cases in 2009–10 were referred to the ICU (3.6% and 4.8% vs. 18.8%, p<0.001) and were classified as DFCS (7.1% and 10.6% vs. 21.2%, p<0.01) or DSAS (1.8% and 2.9% vs. 13.7%, p<0.001; Table 5). Likewise, more DENV-3 cases in 2009–10 presented compensated shock and its associated signs, delayed capillary refill and cold extremities, than in 2005–9 or 2010–11 (p<0.001). Kaplan-Meier survivor curves indicate that these same signs presented significantly earlier in 2009–10 than 2005–9 and 2010–11 (Fig. 6A–C). In Cox regression models controlling for early presentation and immune responses, cases in 2009–10 had increased risk of compensated shock, delayed capillary refill and cold extremities (Table 6). Finally, in multivariate analysis adjusting for age, sex and immune status, year 2009–10 cases had a 2.62 greater risk of developing DFCS compared with cases in 2005–9 or 2010–11 (95% CI 1.25–5.49, p = 0.011) (Table 7). In the cohort, similar trends were seen among cases of DENV-3 in 2009–10 vs. 2010–11 (data not shown), though the small number of cases of DENV-3 in the cohort in 2004–9 precluded a complete analysis as in the hospital study.

**Table 5:** Demographic and clinical characteristics of DENV-3 cases, Hospital Study, 2005–11.

	2005–9	2009–10	2010–11	p-value^3^
**Symptomatic dengue cases, serotype 3**	112	175	104	
**Sex**				0.861
**Female**	58 (51.8)	87 (49.7)	50 (48.1)	
**Male**	54 (48.2)	88 (50.3)	88 (50.3)	
**Age, years (median, IQR)**	7.0 (3.4–9.3)	8.6 (5.2–10.7)	8.5 (5.8–11.6)	0.003
**Immune Response** 1				0.805
**Primary**	61 (56.0)	88 (52.4)	55 (55.6)	
**Secondary**	48 (44.0)	80 (47.6)	44 (44.4)	
**Day of presentation after onset of symptoms (median, IQR)**	5 (4–5)	3 (2–4)	4 (3–4)	<0.001
**Care**				
**Out-patient**	27 (24.1)	22 (12.6)	8 (7.7)	0.002
**In-patient**	81 (72.3)	120 (68.6)	91 (87.5)	0.002
**Intensive care**	4 (3.6)	33 (18.8)	5 (4.8)	<0.001
**Classification**				
**Dengue Fever (DF)**	69 (61.6)	89 (50.9)	63 (60.6)	0.125
**Dengue Fever with Compensated Shock (DFCS)**	8 (7.1)	37 (21.1)	11 (10.6)	0.002
**Dengue w/ Signs Associated w/Shock (DSAS)**	2 (1.8)	24 (13.7)	3 (2.9)	<0.001
**Dengue Hemorrhagic Fever (DHF)**	27 (24.1)	20 (11.4)	25 (24.0)	0.006
**Dengue Shock Syndrome (DSS)**	6 (5.4)	5 (2.9)	2 (1.9)	0.386
**Clinical Signs and Symptoms**				
**Hemorrhagic manifestations** 2	90 (80.4)	148 (84.6)	83 (79.8)	0.514
**Mucosal bleeding**	18 (16.1)	32 (18.3)	13 (12.5)	0.446
**Positive tourniquet test**	49 (43.7)	102 (58.3)	42 (40.4)	0.006
**Petechiae**	88 (78.6)	136 (77.7)	79 (76.0)	0.896
**Rash**	107 (95.4)	162 (92.6)	101 (97.1)	0.276
**Platelets ≤100,000/mm^3^**	51 (45.5)	46 (26.3)	38 (36.5)	0.003
**Leukopenia (WBC ≤5,000/mm^3^)**	86 (76.8)	162 (92.6)	94 (90.4)	<0.001
**Cold extremities**	32 (28.6)	92 (52.6)	39 (37.5)	<0.001
**Capillary refill >2 seconds**	19 (17.0)	74 (42.3)	24 (23.1)	<0.001
**Compensated shock**	18 (16.1)	71 (40.6)	24 (23.1)	<0.001

1 In the hospital study, immune response is known in 109, 168, and 99 DENV-3 cases in years 2008–9, 2009–10, and 2010–11, respectively.

2 Hemorrhagic manifestations do not include laboratory values and are defined as presence of any of the following clinical signs and symptoms: petechiae, rash, positive tourniquet test, bruising, hematoma, hemoptysis, epistaxis, gingivorrhagia, melena, hematemesis, hematuria, subconjunctival hemorrhage, vaginal hemorrhage, hypermenorrhea and excessive bleeding at puncture site.

3 p-values were calculated using the Chi-square tests, except for mean age and mean day of presentation, for which Mann-Whitney t-tests were applied.

**Table 6:** Year 2009–10 as most significant risk factor in Cox regression models of compensated shock, DENV-3 cases, Hospital Study, 2005–11.

	Hazard Ratio for Year 2009–10^1^	95% CI	p-value
**Cold extremities**	2.15	1.39–3.14	<0.001
**Capillary refill >2 seconds**	2.85	1.64–4.93	<0.001
**Compensated shock**	2.77	1.60–4.80	<0.001

1 Cox regression models were created to determine risk factors associated with “compensated shock”, poor capillary refill, and cold extremities in DENV-3 cases from the Hospital study, controlling for dengue season (2009–10 and 2010–11, with 2005–9 as reference), immune response (primary versus secondary DENV infection), age (<5 versus ≥5 years old), sex, and early presentation (≤3 days versus >3 days since onset of symptoms). Year 2009–10 emerged as the most significant risk factor in all models, with values as indicated.

**Table 7:** Relative risk of DFCS and DFCS/DSS/DSAS in 2009–10 in DENV-3 cases, Hospital Study.

	Relative Risk^1^	95% CI	p-value
**DFCS vs. All other dengue**	2.62	1.25–5.49	0.011
**DFCS vs. DF**	2.37	1.17–4.81	0.017
**DFCS/DSS/DSAS vs. DF/DHF**	2.54	1.48–4.40	0.001

1 Relative risk for the events are adjusted for dengue season (2009–10 and 2010–11, with 2008–9 as reference), immune response (primary versus secondary DENV infection), age (<5 versus ≥5 years old), sex, and early presentation (≤3 days versus >3 days since onset of symptoms). Year 2009–10 emerged as the only significant risk factor in all models, with values as indicated.

### 2009 Influenza A H1N1 Pandemic

**Figure 7:**
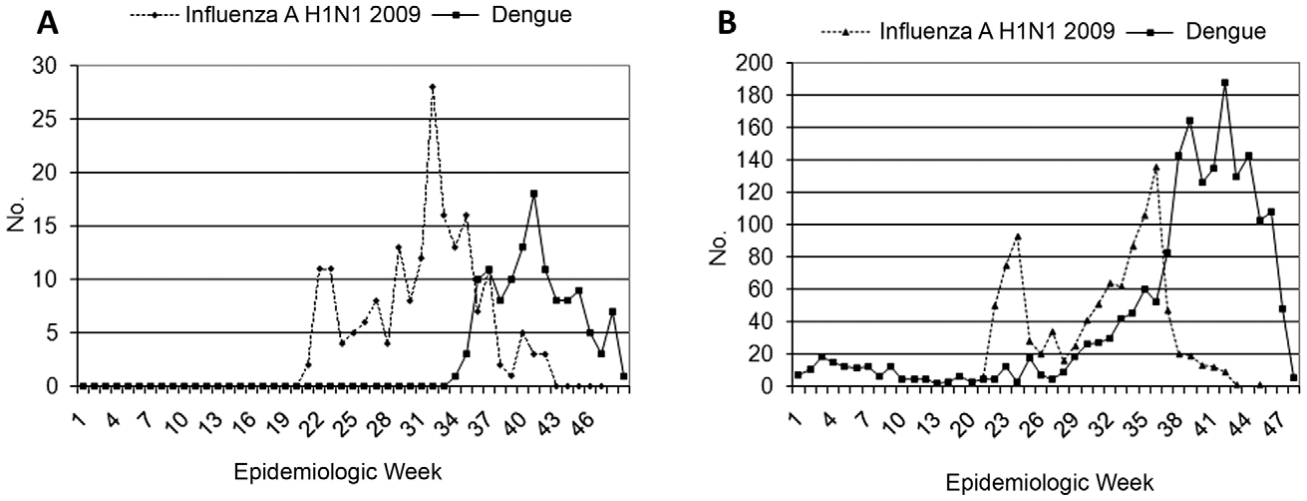
Epidemiologic curves of dengue and Influenza A H1N1-2009 in the cohort study and Nicaragua, 2009. *A*, Influenza A H1N1 and dengue cases in the cohort study by week, 2009. *B*, Influenza A H1N1 and dengue cases in Nicaragua by week, 2009. National surveillance statistics for influenza (CNDR/Ministry of Health) were used to determine the cases of Influenza H1N1 2009 by week in 2009.

The atypical, early onset of compensated shock and poor peripheral perfusion we observed is unique to the 2009–10 dengue season, when neither DENV serotype nor clade changed. Our leading hypothesis for what may have caused this unusual clinical presentation is the concurrent circulation of pandemic Influenza A during the 2009 dengue season, the only notable epidemiological distinction that occurred in 2009. Usually, influenza peaks in June–July [26], 2–3 months before the beginning of the annual dengue season. However, in 2009, although Influenza A H1N1 virus began circulating in Managua in June, the influenza pandemic was more prolonged that year [26]; incidence reached a maximum in August and overlapped for 8–10 weeks with the 2009–10 dengue epidemic (Fig. 7A and B). One hypothesis is that recent infection with Influenza A H1N1 2009 might have modulated the immune response to a subsequent DENV infection. Therefore we tested for the presence of anti-H1N1 2009 antibodies in 2009–10 hospital dengue cases. In serum samples from 187 DENV-positive subjects in the hospital study in 2009–10, 65.7% (123) contained antibodies specific to the Influenza A H1N1 2009 strain, indicating recent infection with the virus. Subjects with H1N1 antibodies had significantly greater odds of developing compensated or hypotensive shock (DFCS, DSAS or DSS) compared with subjects without H1N1 antibodies (43% vs. 28%, respectively; OR 1.93, 95% CI 1.01–3.31; p = 0.045).

## Discussion

In this paper, we have described an unusually large dengue epidemic with a unique clinical presentation characterized by early presentation of poor peripheral perfusion. In our pediatric cohort study, we observed incidence rates more than twice as high as in previous study years (4.6% vs. <2%), a finding that reflected observations in our parallel hospital-based study. Both the epidemiologic and clinical trends reported in this study were also observed by national authorities and reflected in the national surveillance statistics for the 2009–10 dengue season in Managua. This epidemic was associated with atypical presentation of dengue, with signs of poor peripheral perfusion presenting 1–2 days earlier than usual in the course of illness and less progression to DHF/DSS in both studies. The usual evolution of dengue toward severe disease involves rising hematocrit and falling platelet count followed by onset of compensated shock leading to hypotensive shock (hypotension for age or narrow pulse pressure) accompanied by clinical signs of shock (e.g., poor capillary refill, cold extremities). This latter is termed the critical phase and generally occurs on days 4–5 (range 3–7) after onset of symptoms [2], [27].

Compensated shock had not previously been seen on a large scale in Nicaragua, nor has it been reported elsewhere as a defining clinical feature of dengue without progression to hypotensive shock. In 2009–10, study physicians treated 96% of subjects presenting “compensated shock” with IV therapy compared to 83% in previous study years. Those cases that did not respond to crystalloid fluid therapy were administered colloids, and if still no improvement was seen, patients were transferred to the ICU, and if necessary, administered amine vasopressor agents. Only 31% of those administered crystalloids were not responsive, suggesting that such therapy limited progression to more severe disease. The recently revised WHO guidelines for dengue management suggest treatment of compensated shock with crystalloids [27]. The early presentation of compensated shock in the 2009–10 epidemic coupled with early IV fluid administration may well have enhanced the effect of IV fluid treatment on the course of disease.

DENV serotype and immune response have been linked to variations in clinical presentation of dengue. However, in this study, in multivariate analysis controlling for serotype, immune response, sex, and age less than 5 years, presenting in 2009–10 was the most significant risk factor for DFCS compared with uncomplicated dengue or all other dengue outcomes, and similar results were obtained with DFCS, DSAS, and DSS compared to dengue without shock. Presenting in 2009–10 was also the most significant risk factor for early presentation of signs of poor peripheral perfusion and compensated shock in Cox regression models. DENV-3 was responsible for the majority of cases in the 2008–9 and 2009–10 seasons, with no change in frequency or viral sequence. Another large dengue epidemic during the 2010–11 season was dominated by the same clade of DENV-3, yet clinical presentation was once again typical and involved neither high frequencies nor early presentation of “compensated shock” as in 2009–10. In addition, the sequence of DENV infections and immune status appear not to be factors in the unique clinical presentation of DENV-3 in 2009–10. In the hospital, where more severe secondary cases are typically seen, 2009–10 had significantly more primary infections than previous study years (p<0.001) and similar frequencies of primary cases as in 2008–9 – consistent with the higher proportion of primary cases often observed with DENV-3 [3], [4], [9]. This suggests that DENV immune response did not impact severity, as the clinically severe cases of 2009–10 were just as likely to be primary as secondary DENV infections. Lastly, multivariate analysis ruled out immune status (i.e., secondary infection and thus sequence of DENV serotypes) as responsible for the unusual clinical characteristics of dengue in 2009–10.

Thus, our data indicate that the distinct disease phenotype observed in 2009–10 was not due to DENV serotype, viral sequence, immune status, or serotype sequence of sequential DENV infections and lends support to the hypothesis that something about the year 2009–10 was different. In addition, the ratio of symptomatic to inapparent DENV infections increased in 2009–10 (1∶1.2) relative to 2008–9 (1∶9.5), implying that the overall incidence of symptomatic DENV infection was substantially higher in 2009–10. While early IV fluid intervention and increased hospitalization may have affected disease progression, decreasing plasma leakage and other typical symptoms of severe disease in 2009–10, case management could not have affected the early presentation of the symptoms of poor peripheral perfusion in the first place, since the children arrived at the hospital with these signs and symptoms prior to any interventions. Currently, our favored hypothesis is that there may be an interaction between a recent Influenza A H1N1 2009 infection and the subsequent DENV infection, as high rates of Influenza A H1N1 2009 in the cohort and in Managua as well as a number of identified DENV/H1N1 2009 co-infections [28] suggest that concurrent or previous H1N1-2009 infection may have influenced the atypical clinical presentation of dengue cases in 2009–10. The influenza pandemic occurred later than usual (August–September rather than June–July) in 2009–10 and thus preceded/overlapped the annual dengue epidemic in an unusual way that is not normally seen in Nicaragua [26], evidenced in both the cohort and the national surveillance system. We posit that a prior influenza infection may be modulating the subsequent immune response to DENV infection, perhaps by reducing interferon levels [29], which could lead to greater DENV infection, as it is known that type I interferons are powerful antagonists of DENV infection [30], [31]. Influenza infections are known to predispose towards and enhance the severity of secondary bacterial infections [32], [33], [34], [35], [36]; perhaps in close temporal proximity, a similar effect could occur on a subsequent DENV infection. To investigate this hypothesis, a case control study is underway in which acute samples from 2009 dengue cases in the cohort and hospital studies with or without compensated/hyptotensive shock are being tested for the presence of antibodies specific to the Influenza A H1N1 2009 strain. Initial analyses presented here support this hypothesis. Cytokine profiles and viremia in these samples are also being investigated. In addition, we are establishing a mouse model of influenza virus followed by DENV infection to explore the existence and then mechanistic underpinnings of this proposed immunomodulation.

In summary, the 2009–10 dengue epidemic in Managua was large and involved an atypical presentation of the disease, with early onset of signs of poor peripheral perfusion (“compensated shock”), as observed in both long-term community-based and hospital studies. Multivariate analysis controlling for usual risk factors associated with severe dengue revealed only study year 2009–10 as a significant risk factor for DFCS. Another unusual feature of 2009 in Managua was the circulation of pandemic influenza A H1N1, which for the first time overlapped with the dengue season. We postulate that the unusual presentation of dengue in 2009 may have been due in part to immunomodulation by a prior influenza H1N1-2009 infection and are currently testing this hypothesis. Overall, this study demonstrates that parameters other than DENV serotype, viral genomic sequence, immune status, and sequence of serotypes can play a role in modulating dengue disease outcome.
